# Real-Time Detection and Monitoring of Oxide Layer Formation in 1045 Steel Using Infrared Thermography and Advanced Image Processing Algorithms

**DOI:** 10.3390/ma18050954

**Published:** 2025-02-21

**Authors:** Antony Morales-Cervantes, Héctor Javier Vergara-Hernández, Edgar Guevara, Jorge Sergio Téllez-Martínez, Gerardo Marx Chávez-Campos

**Affiliations:** 1 División de Estudios de Posgrado e Investigación, TecNM-Instituto Tecnológico de Morelia, Maestría en Ciencias en Ingeniería Electrónica (MCIE), Av. Tecnológico 1500, Morelia 58120, Mexico; antony.mc@morelia.tecnm.mx (A.M.-C.); hector.vh@morelia.tecnm.mx (H.J.V.-H.); jorge.tm@morelia.tecnm.mx (J.S.T.-M.); 2 Faculty of Science, Universidad Autónoma de San Luis Potosí, San Luis Potosi 78294, Mexico; edgar.guevara@uaslp.mx; 3 Coordinación para la Innovación y Aplicación de la Ciencia y la Tecnología (CIACYT), Universidad Autónoma de San Luis Potosí, San Luis Potosi 78210, Mexico

**Keywords:** high-temperature oxidation, AISI 1045 steel, infrared thermography, image processing algorithms, mechanical properties, phase stability, steel manufacturing

## Abstract

This study addresses the challenge of monitoring oxide layer formation in 1045 steel, a critical issue affecting mechanical properties and phase stability during high-temperature processes (900 °C). To tackle this, an image processing algorithm was developed to detect and segment regions of interest (ROIs) in oxidized steel surfaces, utilizing infrared thermography as a non-contact, real-time measurement technique. Controlled heating experiments ensured standardized data acquisition, and the algorithm demonstrated exceptional accuracy with performance metrics such as 96% accuracy and a Dice coefficient of 96.15%. These results underscore the algorithm’s capability to monitor oxide scale formation, directly impacting surface quality, thermal uniformity, and material integrity. The integration of thermography with machine learning techniques enhances steel manufacturing processes by enabling precise interventions, reducing material losses, and improving product quality. This work highlights the potential of advanced monitoring systems to address challenges in industrial steel production and contribute to the sustainability of advanced steel materials.

## 1. Introduction

Steel production has increased considerably in recent years [1]. This growth has driven the optimization of processes to obtain better quality products [2]. However, challenges such as decarburization and oxidation remain prevalent in steel plants [3]. Metals heated to high temperatures develop an oxide layer on the surface, leading to changes in the structure and properties of the metal [4]. The thermal conductivity of oxide layers is significantly lower than that of steel and varies linearly with temperature. Additionally, the emissivity of steel surfaces plays a critical role in thermal radiation analysis during high-temperature processes [5]. These layers form when the material is heated above 593 °C [6]. Factors such as the heating time, atmospheric oxygen potential, and carbon chemical potential gradient are determinant for the degree of oxidation and decarburization of steel [7,8]. The growth of oxide layers directly impacts the thermal and mechanical properties of steel, influencing uniformity, strength, and surface quality. Controlling these oxide layers is critical to minimize material losses and optimize the performance and durability of advanced steel components. [9] Therefore, maintaining a controlled environment during the heating and cooling processes of the metal can improve its quality [10].

Various techniques and instruments have been employed to enhance steel heating processes and product quality, including optical microscopy [2] and automated vision-based inspection techniques [11]. Recent studies have explored the use of infrared thermography as a non-contact method to monitor surface reactions and their effects on steel solidification under different gas atmospheres, highlighting its potential in characterizing surface phenomena in steel processing [12]. Monitoring the surface temperature of the material can reveal changes occurring in the steel as oxide layers form. Infrared thermography has been applied in real-time quality monitoring of laser cladding processes on rail steel, demonstrating its effectiveness in detecting discrepancies in cladding conditions and its potential for process optimization [13]. The application of thermography in monitoring oxide layer formation can contribute to improving process efficiency and final product quality [12].

Recent studies have demonstrated the effectiveness of unsupervised learning-enabled pulsed infrared thermographic microscopy for detecting subsurface defects in stainless steel, highlighting its potential for oxide monitoring in industrial applications [14]. Additionally, cooling-excited infrared thermography has been employed to enhance defect detection in steel–concrete interfaces, demonstrating improved sensitivity to interfacial oxidation processes [15]. In steel structures, thermographic inspection methods, particularly pulsed thermography, have been utilized for analyzing internal defects and oxidation layers with advanced signal processing techniques [16]. Beyond thermography, Raman spectroscopy has been widely used to characterize oxide scales, offering detailed phase identification of corrosion products and high-temperature oxidation layers in stainless steels [17,18].

Infrared thermography is a technique that produces an image of the temperature distribution on a surface [19]. Thermal cameras have sensors that capture the thermal radiation emitted or reflected by objects. The thermal images generated by these cameras, called thermograms, assign a digital value to each pixel proportional to the amount of electromagnetic energy received [20]. This technique is utilized in various fields, including industry, military, construction, and medicine [21]. All objects with a temperature above absolute zero emit electromagnetic radiation, known as infrared radiation or thermal radiation [22,23,24]. The wavelength of this radiation ranges from 0.75 to 1000 μm, subdivided into near-infrared (NIR: 0.76–1.5 μm), mid-infrared (MIR: 1.5–5.6 μm), and far-infrared (FIR: 5.6–1000 μm). According to the theory of thermal radiation, a black body is a hypothetical object that absorbs all incident radiation and emits a continuous spectrum according to Planck’s law [24]. Integrating Planck’s law over all frequencies yields the Stefan–Boltzmann law Equation (1), which describes the total emission irradiance of a black body [24].

(1) $$E=\sigma T^{4}$$

where *E* is the emissive irradiance (${W/m}^{2}$), σ is the Stefan–Boltzmann constant ($\sigma =5.676\times 10^{-8}\;{W/m}^{2}\;K^{4}$), and *T* is the absolute temperature (K). For real surfaces, the Stefan–Boltzmann constant is modified as follows:

(2) $$E=\epsilon \sigma T^{4}$$

where ϵ is the surface emissivity at a given wavelength and absolute temperature *T*. For an ideal emissivity, as in a black body, ϵ equals unity, but for real materials, the emissivity is always less than unity. The emissivity range for steel is 0.5–0.85 [25].

This article contributes to the understanding of oxide layer behavior in 1045 steel, focusing on how real-time monitoring and control of these layers can enhance the mechanical properties and phase stability of steel during high-temperature processes. The proposed method utilizes temperature differences over the region of interest (ROI), corresponding to the oxide layer forming on the sample surface.

This study presents a novel approach by integrating infrared thermography with advanced image processing techniques to detect and monitor oxide layer formation in 1045 steel in real time. Unlike previous research that primarily focused on post-process analysis using optical microscopy or destructive testing methods, this work enables in situ monitoring of oxidation. The proposed algorithm not only enhances detection accuracy but also introduces an automated segmentation process that minimizes human intervention. Additionally, this methodology is adaptable for real-time industrial applications, offering a non-destructive, contactless, and precise alternative for steel oxidation monitoring.

## 2. Materials and Methods

The experimental part was carried out in two principal stages: the heating and data acquisition stage and the image acquisition and processing stage. This study aimed to implement and evaluate an algorithm for detecting oxide layers on 1045 steel specimens using infrared thermography.

### 2.1. Experimental Setup

**Figure 1:**
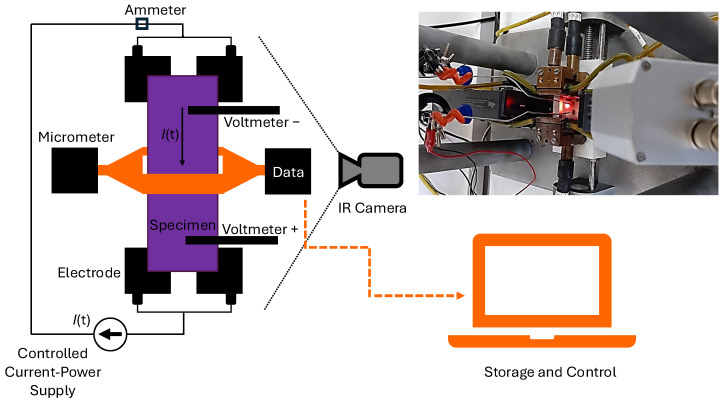
Configuration of the experiment for the acquisition of images by infrared thermography.

High-temperature oxidation experiments were conducted on cylindrical 1045 steel specimens with a diameter of 3.8 mm and a length of 26 mm. The oxidation process was carried out in a custom-designed controlled Joule heating system. The experiment lasted a total of 50 min, with temperatures exceeding 900 °C. The heating of the specimen was performed incrementally, with the current increased by 16 amperes every minute until reaching 160 amperes. This current was maintained for 30 min to analyze the surface changes induced by the oxide layer formation. Finally, the specimen was cooled down gradually, decreasing the current by 16 amperes every minute until reaching 0 amperes. The setup for infrared image acquisition during the heating of 1045 steel is depicted in Figure 1.

The thermal images were captured using an Optris PI 1M (Optris GmbH, Berlin, Germany) digital infrared camera equipped with a microbolometer-based focal plane array. The camera has a thermal sensitivity of <1 K (700 °C) and <2 K (1000 °C), an accuracy of ±1%, a spectral range of 0.85–1.1 μm, and a temperature range of 450–1800 °C. This thermal imaging system provides real-time thermal data with a resolution of 764 × 480 pixels, which can be stored digitally. Measurements were taken with a constant emissivity of 0.8 [25].

**Figure 2:**
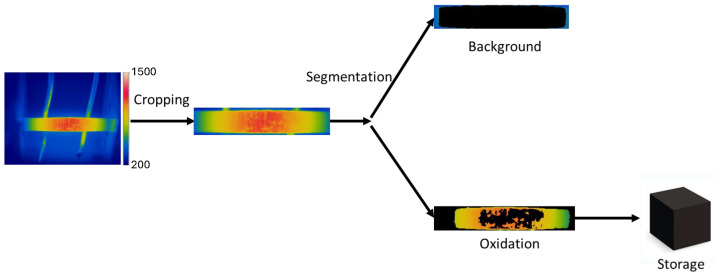
Flowchart of the image processing performed.

Digital image processing was performed using Python 3.10.14, Anaconda distribution. The preprocessing stage involved the selection of the region of interest (ROI) and the extraction of areas with oxidation from the material. The overall image processing workflow is shown in Figure 2.

The literature has demonstrated that the oxidation of 1045 steel at high temperatures leads to the formation of distinct oxide phases, including Fe_1−*x*_O (wüstite), Fe_3_O_4_ (magnetite), and Fe_2_O_3_ (hematite), each with specific properties that contribute to the overall behavior of the oxide layer [26]. The sequential formation and coexistence of these phases depend on temperature, oxygen availability, and exposure time, making them critical factors in the oxidation process.

### 2.2. Material Thickness Limitations in Infrared Thermography

The effectiveness of infrared thermography for monitoring material thickness is limited by the thermal and optical properties of the material. For 1045 steel, the penetration depth of infrared radiation is shallow due to its high absorption and low infrared transparency in the mid-wave infrared (MWIR: 3–5 µm) and long-wave infrared (LWIR: 8–14 µm) spectral ranges.

Infrared thermography is primarily a surface measurement technique, with its effectiveness in depth monitoring governed by the thermal diffusion length (δ) given by

(3) $$\delta =\sqrt{\alpha t}$$

where α is the thermal diffusivity of steel ($12\;{\mathrm{mm}}^{2}/s$), and *t* is the characteristic heating time. Under steady-state conditions, infrared thermography mainly provides surface temperature measurements.

Previous studies have demonstrated that infrared thermography in steels can effectively detect surface and near-surface temperature variations within a depth range of approximately 0.1–1 mm [12,23].

### 2.3. Image Processing Methods

Image segmentation is a technique used to identify relevant information in digital images by dividing the main image into different segments. The method used for specimen segmentation in this study is based on the projection profile [27]. This method identifies the top, bottom, left, and right edges of the thermogram by counting the number of black and white pixels in each row and column. The sequence of operations for processing the thermographic image is detailed in Figure 2.

#### 2.3.1. Selection of Material Area with Sobel Operator

The Sobel operator measures the 2D spatial gradient of the thermal image, emphasizing regions of high spatial frequency that correspond to edges. It consists of a pair of 3 × 3 masks designed to detect edges vertically and horizontally. The Sobel operator smooths the data, reducing false edges [28]. The gradient of the image f(x,y) is a vector given by

(4) $$\nabla =\left[\begin{array}{c} G_{x}\\ G_{y} \end{array}\right]=\left[\begin{array}{c} \frac{\partial f}{\partial x}\\ \frac{\partial f}{\partial y} \end{array}\right]$$

The magnitude of the gradient is

(5) $$\left|\nabla f\right|=\sqrt{{G_{x}}^{2}+{G_{y}}^{2}}$$

The direction of the edge is

(6) $$\alpha \left(x,y\right)=\tan^{-1}\frac{G_{y}}{G_{x}}$$

The coefficient matrix for the Sobel operator is

(7) $$\begin{array}{c} H_{x}=\left[\begin{array}{c} -1\;0\;1\\ -2\;0\;2\\ -1\;0\;1 \end{array}\right]\;y\;H_{y}=\left[\begin{array}{c} -1\;-2\;-1\\ 0\;0\;0\\ 1\;2\;1 \end{array}\right] \end{array}$$

The Sobel operator produces estimates of the local gradient for all pixels in the image in two directions, maintaining the following relationship:

(8) $$\nabla I\left(x,y\right)\approx \frac{1}{8}\left[\begin{array}{c} H_{x}\;\cdot \;I\\ H_{y}\;\cdot \;I \end{array}\right]$$

The filter results for each direction are given by

(9) $$D_{x}\left(x,y\right)=H_{x}*I\;y\;D_{y}\left(x,y\right)=H_{y}*I$$

**Figure 3:**
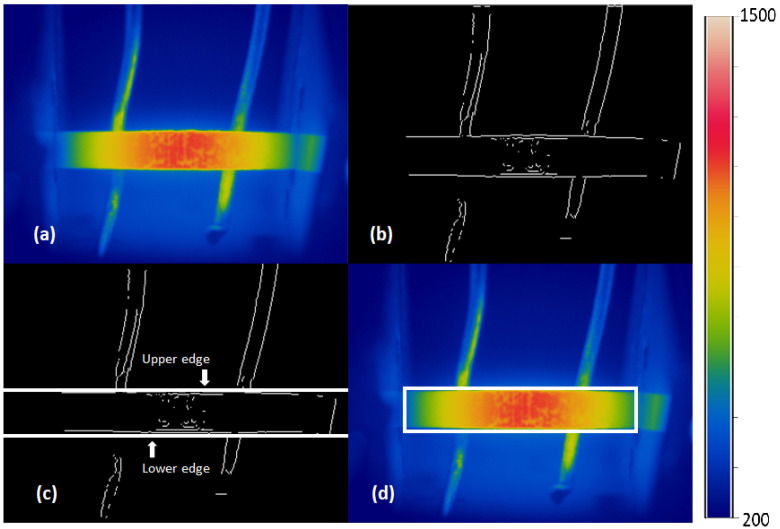
(**a**) Specimen thermogram; (**b**) binarized image with edge detection; (**c**) top and bottom limits detected; and (**d**) left, top, right, and bottom edges detected.

The Figure 3 shows the result of the edge detection of the thermogram. The limits of the area were successfully detected using the Sobel operator, as shown in Figure 3b.

#### 2.3.2. Material Boundary Detection

To identify the edges of the specimen, the upper edge was found first. The detected edges in the image (Figure 3b) were scanned by rows (horizontally), counting the number of white pixels in each row from the top of the image. The scan was repeated until the Horizontal Projection Profile (HPP) value was equal to or greater than the given preset value, which was chosen through testing to detect the upper edge of the specimen. The row number corresponding to the first high HPP value was taken as the upper limit for segmenting the thermographic image of the specimen. The procedure to find the lower limit was carried out similarly, counting the number of white pixels in each row from the bottom of the image. The upper and lower limits detected are shown in Figure 3c.

The thermographic image of the specimen was segmented using the upper and lower limit values, considering only the area below the upper limit and above the lower limit. The left and right edges were detected using the Vertical Projection Profile (VPP) method, which counts the number of white pixels in each column. To find the left boundary, the image was scanned from left to right, and the position of the first white pixel in each column was stored. To find the right limit, the image was scanned from right to left, storing the position of the first white pixel found in each column. These detected edges were used to define the region of interest (ROI) in the thermographic image of the specimen. The final segmented image with highlighted edges is shown in Figure 3d.

#### 2.3.3. Calculation of Thermal Image Oxidation

The process for calculating the oxidized part of the image was carried out in the CIELAB color space, which encompasses all the colors perceivable by the human eye. The CIELAB color space allows visual differences in an image to be quantified. The space is defined by three variables: *L* (lightness), *a* (green-red axis), and *b* (blue-yellow axis) [28].

**Figure 4:**
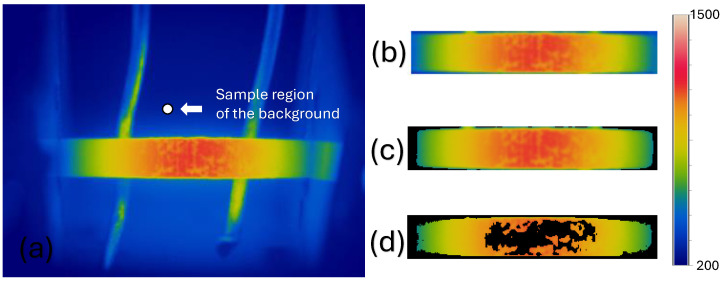
(**a**) Area used to remove background, (**b**) segmented region of interest, (**c**) region of interest with background removed, and (**d**) non-uniform oxidation.

Figure 3d shows the areas with different temperatures in the specimen. During segmentation, the image is first converted from RGB to CIELAB color space. A sample region from the background of the image is selected to obtain *a* and *b* values (Figure 4a). The average *a* and *b* values of the selected area are calculated and saved as markers. Each pixel is then classified by calculating the Euclidean distance between the pixel and the marker. If the distance is minimal, the pixel is labeled as background. A mask image is generated with the classified pixels, which is used to separate the background from the original image.

Once the original image is color-segmented, the preset limits (top, bottom, right, and left) are used to segment the outlines (Figure 4b), and the background is removed from the images (Figure 4c). To find the oxide formation, temperature changes in the ROI are analyzed. Given a thermographic image of m×n pixels, if each pixel of the temperature matrix is represented by $x_{\left(i,j\right)}$, the non-uniform oxide growth classification is given by

(10) $$x_{\left(i,j\right)}\leq {\bar{x}}_{j}$$

where

(11) $${\bar{x}}_{j}=\frac{{\bar{y}}_{j-1}+{\bar{y}}_{j}+{\bar{y}}_{j+1}}{3}\;\forall \;j=\left\{2,3,\ldots ,n-1\right\}$$

and

(12) $$\bar{y}=\frac{1}{m}\sum_{i=1}^{m}x_{i}=\frac{x_{1}+x_{2}+\ldots +x_{m}}{m}$$

Finally, the temperature changes recorded in the ROI are extracted (Figure 4d) for further analysis.

### 2.4. Evaluation Metrics

To assess the performance of the proposed algorithm, standard metrics for image segmentation were used. These include precision, recall, F1-score, Dice coefficient, intersection over union (IoU), true negative rate (TNR), and accuracy. These metrics provide a comprehensive evaluation of the algorithm’s ability to correctly identify the presence and absence of oxide layers. The definitions and formulas for these metrics are as follows:

Precision: Measures the proportion of true positive identifications relative to the total positive identifications made by the algorithm.

(13) $$\mathrm{Precision}=\frac{TP}{TP+FP}$$

Recall: Measures the proportion of true positive identifications relative to the total actual positives.

(14) $$\mathrm{Recall}=\frac{TP}{TP+FN}$$

F1-score: The harmonic mean of precision and recall, providing a balance between the two.

(15) $$F1-\mathrm{Score}=2\;\cdot \;\frac{\mathrm{Precision}\;\cdot \;\mathrm{Recall}}{\mathrm{Precision}+\mathrm{Recall}}$$

Dice coefficient: A measure similar to the F1-score used to gauge the similarity between two sets.

(16) $$\mathrm{Dice}\;\mathrm{Coefficient}=\frac{2\;\cdot \;TP}{2\;\cdot \;TP+FP+FN}$$

IoU (intersection over union): Measures the overlap between the predicted segmentation and the ground truth.

(17) $$\mathrm{IoU}=\frac{TP}{TP+FP+FN}$$

True negative rate (TNR): Measures the proportion of true negative identifications relative to the total actual negatives.

(18) $$TNR=\frac{TN}{TN+FP}$$

Accuracy: Measures the proportion of true identifications (both positive and negative) relative to the total number of cases.

(19) $$\mathrm{Accuracy}=\frac{TP+TN}{TP+TN+FP+FN}$$

where TP (true positive) represents the number of correctly identified positive pixels, FN (false negative) is the number of positive pixels that were incorrectly identified as negative, FP (false positive) is the number of negative pixels that were incorrectly identified as positive, and TN (true negative) is the number of correctly identified negative pixels. These metrics collectively help in evaluating the performance of the segmentation algorithm in accurately identifying the oxide layer.

## 3. Results and Discussion

**Figure 5:**
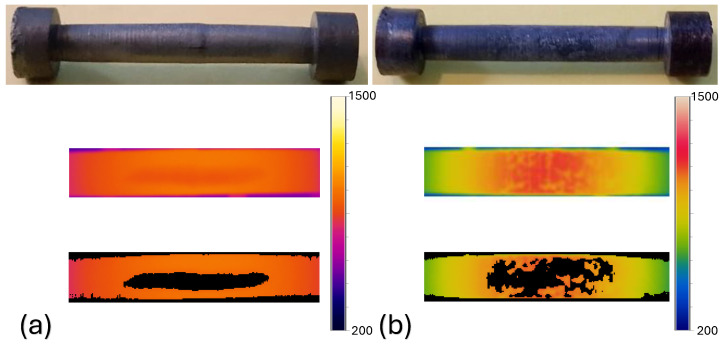
Specimens at the end of the experiments and thermograms taken during heating with (**a**) iron-type color palette and (**b**) rainbow-type color palette.

**Figure 6:**
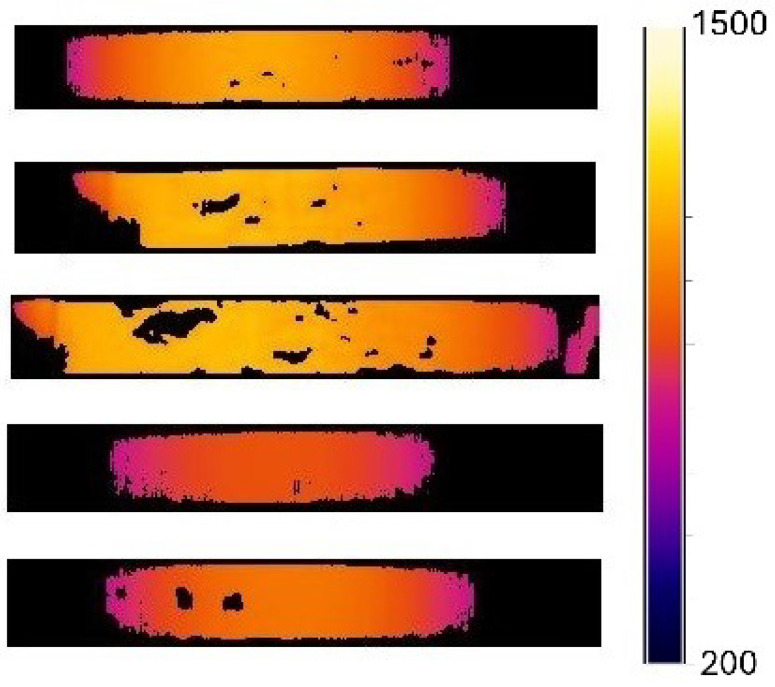
Oxide layer detection applied to different experiments.

Twenty heating experiments were performed on 1045 steel specimens. During data acquisition, different color palettes were tested for the thermographic images taken, of which the “iron” type (Figure 5a) and “rainbow” (Figure 5b) were the ones that best showed the oxide formed on the surface of the specimen. These palettes were chosen based on their ability to visualize oxidation patterns effectively. The iron palette highlights subtle variations in oxidation by emphasizing warm-to-cool transitions, making it particularly effective for identifying temperature-induced changes in steel. Meanwhile, the rainbow palette aligns with human visual perception, improving interpretability. Since the image processing method relies on segmentation in the HSB color space, these palettes also facilitate a more accurate separation of oxidized and non-oxidized regions based on hue and saturation differences. Figure 5 illustrates the effectiveness of these palettes in detecting oxidation patterns. Currently, quality verification processes in steel products are mainly conducted using optical microscope techniques, applying standards to estimate the depth of decarburization in steel samples [29]. There is great potential for integrating new methods that allow real-time monitoring of the evolution of the solid layer during the casting of steel, heat transfer during solidification, and the types of surface oxides that form on the material [12]. Although algorithms and techniques based on infrared thermography are currently being investigated to improve processes in steel manufacturing, it remains a poorly explored topic with ample potential for future research [30]. To provide a comprehensive analysis of the oxidation process, additional thermographic images were captured at different time intervals, showing the evolution of the oxide layer over time. These images reveal that the oxidation was not uniform across the specimen, with regions experiencing varying degrees of oxidation depending on temperature gradients and surface conditions. The segmentation results applied to different heating experiments are shown in Figure 6, highlighting areas of higher oxidation intensity.

The first notable point observed during the experiments is that a sudden increase in temperature produced a greater amount of non-uniform oxide in the material. This effect is visually evident in Figure 5, where Figure 5b corresponds to a specimen that experienced a rapid temperature increase, leading to a more heterogeneous oxide distribution. In contrast, Figure 5a represents a specimen with a more gradual heating rate, resulting in a more uniform oxide layer. These observations suggest that the formation and distribution of oxide are strongly influenced by temperature gradients, which may play a crucial role in the oxidation process of steel. Another relevant point is that the growth of the oxide layer can be monitored throughout the process, which is advantageous for implementing estimators for better process control. Finally, this study demonstrates the ability of using thermography and the implementation of the image processing techniques described in the methodology to obtain information about the parts where there is no uniform formation of oxide. This allows for an understanding how the oxide scales are formed during the entire process of the experiment. More experiments were conducted under the same conditions to subsequently apply the proposed algorithm to check the repeatability of the described image processing. To validate both the repeatability and accuracy of the proposed image processing algorithm, a rigorous validation process was conducted. After the heating experiments, visual inspections of the specimens were performed to confirm the presence of oxidation in the regions highlighted by the thermographic images. Thermograms were analyzed alongside post-experiment observations, where the oxidized areas were manually segmented by domain experts in thermography and metallurgy. These manually segmented masks served as a reference standard for evaluating the algorithm’s segmentation performance. The algorithm’s output was assessed using a confusion matrix, comparing the detected oxide areas with the manually segmented regions. Across multiple trials, the algorithm consistently demonstrated high accuracy in detecting oxidation patterns, with minimal variance, confirming its robustness and repeatability for real-time monitoring in industrial applications.

**Figure 7:**
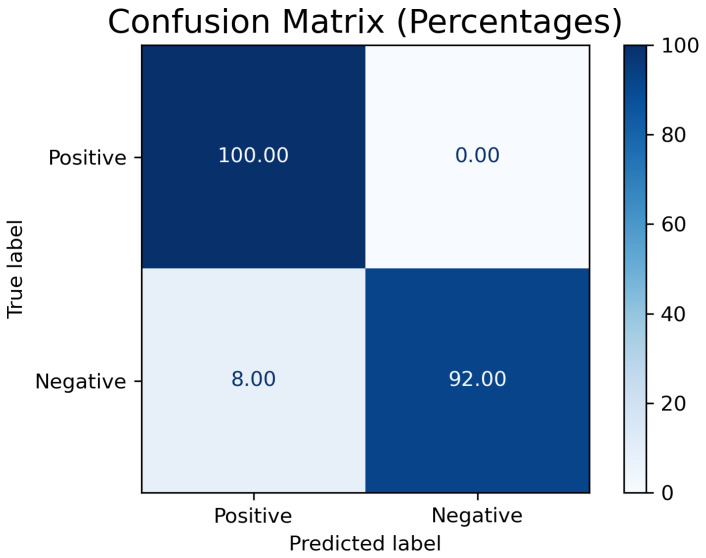
Confusion matrix for the oxide layer segmentation algorithm.

The performance of the proposed algorithm was evaluated using standard metrics for image segmentation. The results indicate that the algorithm achieved high performance in accurately identifying and segmenting the oxide layer on the steel surface. The evaluation metrics obtained are the average results from the analysis of all experiments and are summarized in Table 1. Additionally, the confusion matrix in Figure 7 illustrates the distribution of true positives, true negatives, false positives, and false negatives. The performance of the proposed algorithm was evaluated using standard metrics for image segmentation. The results indicate that the algorithm achieved a precision of 92.59%, a recall of 100%, an F1-score of 96.15%, a Dice coefficient of 96.15%, an intersection over union (IoU) of 92.59%, a true negative rate (TNR) of 92%, and an accuracy of 96%. These metrics demonstrate the algorithm’s effectiveness in accurately identifying and segmenting the oxide layer on the steel surface. A closer examination of the precision and recall values indicates that the algorithm effectively minimized false negatives, ensuring that most oxidized regions were detected. However, the presence of some false positives suggests that certain areas with high temperature variations but without significant oxidation may be incorrectly classified as oxidized. To address this, future work should explore the integration of additional texture-based features or machine learning classifiers to refine segmentation accuracy.

The high recall value of 100% indicates that the algorithm is highly sensitive and correctly identified all the pixels that belong to the oxide layer. The precision value of 92.59% shows that the majority of the pixels identified as oxide were indeed correct, although there wer some false positives. The F1-score and Dice coefficient, both at 96.15%, reflect a strong balance between precision and recall, indicating robust overall performance.

The true negative rate (TNR) of 92% suggests that the algorithm effectively distinguished non-oxide areas, minimizing false positives. The accuracy of 96% demonstrates the overall effectiveness of the algorithm in correctly classifying both oxide and non-oxide pixels.

These results highlight the potential of using infrared thermography combined with advanced image processing algorithms for real-time monitoring and quality control in steel manufacturing. One important consideration for future applications of this method is the adaptation to three-dimensional surfaces with complex geometries. Since infrared thermography relies on the accurate measurement of emitted radiation, variations in surface orientation and curvature can introduce errors due to changes in emissivity and reflection angles. In practical scenarios, this could be mitigated by using structured light techniques or multi-angle thermal imaging to reconstruct the surface geometry and compensate for distortion effects [31]. Future research should focus on integrating 3D surface reconstruction algorithms with thermographic imaging to enhance accuracy in complex geometries. The ability to accurately detect and segment oxide layers can lead to improved process control, reduced material losses, and enhanced product quality. Future research can explore the integration of this algorithm with real-time monitoring systems and the application of machine learning techniques to further improve segmentation accuracy and efficiency.

Recent studies have explored different image processing techniques for detecting oxidation and corrosion in metal surfaces. One study proposed a computer vision system for detecting residual oxidation on stainless steel surfaces in an annealing and pickling line. This approach employs image processing and segmentation techniques to identify oxidized regions, achieving detection and classification accuracy levels ranging from 68% to 98%, depending on the sample analyzed. This highlights the variability in the effectiveness of vision-based methods for oxidation detection in industrial applications [32]. Another recent study applied three deep learning-based semantic segmentation models FCN, U-Net, and Mask R-CNN for corrosion detection on metal structures. While deep learning approaches improve detection accuracy compared to traditional methods, they still struggle with boundary definition, often requiring additional post-processing. The best-performing model achieved a precision of approximately 85% after integrating a boundary refinement technique [33]. Compared to these approaches, our method leverages infrared thermography combined with an HSB-based segmentation algorithm. This approach not only achieved higher segmentation accuracy (precision of 92.59% and F1-score of 96.15%) but also demonstrated robustness and repeatability across multiple trials. Furthermore, unlike deep learning models that often require large annotated datasets for training, our method provides an efficient, real-time solution for industrial oxidation monitoring without extensive manual annotation or additional post-processing.

Beyond image processing techniques for oxidation detection, infrared thermography also plays a broader role in predictive maintenance strategies for industrial applications. One of the emerging uses of infrared thermography is within Condition-Based Monitoring (CBM), where thermal imaging can serve as a non-contact diagnostic tool for detecting anomalies related to thermal behavior and structural integrity in metallic components [34]. While this study primarily focuses on oxidation detection in steel 1045, the proposed methodology could potentially be integrated into CBM frameworks for predictive maintenance, reducing material degradation and optimizing process efficiency. The ability of infrared thermography to provide continuous, in situ monitoring of oxidation patterns can support decision making in maintenance scheduling, particularly in high-temperature environments where material degradation is a critical concern. Furthermore, integrating infrared thermography with machine learning models for automated anomaly detection has demonstrated promising results in CBM applications, suggesting that future implementations could enhance early fault detection capabilities. However, while CBM approaches often rely on multi-sensor fusion strategies that incorporate vibration analysis, acoustic emissions, and spectroscopic methods, infrared thermography remains a valuable tool due to its non-contact nature and rapid data acquisition.

### Advantages and Limitations

The proposed method presents several key advantages. First, it enables real-time monitoring, allowing for immediate detection of non-uniform oxide growth, which is a significant improvement over traditional post-process analysis techniques. Second, the automated segmentation and detection process improves reproducibility and reduces errors introduced by human subjectivity. Additionally, the use of thermography as a non-contact measurement method enhances safety in high-temperature environments.

However, the technique has certain limitations. One of the primary challenges is the potential effect of surface emissivity variations, which may introduce minor inaccuracies in temperature readings. Moreover, while the method performs well on flat surfaces, additional calibration may be required for complex geometries, as discussed earlier. Additionally, to address the limitations of infrared thermography, a well-established method for chemical and structural characterization of oxides, such as Raman spectroscopy, could be considered. Raman spectroscopy offers advantages such as molecular fingerprinting, high spectral resolution, and sensitivity to different oxide phases. Unlike infrared thermography, which is limited to thermal and emissivity-based detection, Raman spectroscopy can provide direct chemical identification of oxide species, even at submicrometer thicknesses. However, Raman techniques are point-based rather than full field, require intense laser illumination, and may be affected by fluorescence from certain oxide layers.

Future research should focus on compensating for emissivity variations, extending the applicability to three-dimensional surfaces through improved image processing algorithms and sensor fusion techniques, and explore the integration of infrared thermography with Raman spectroscopy or other spectroscopic techniques, enabling a more comprehensive analysis of oxide layer formation in steel manufacturing. Additionally, infrared thermography-based oxidation monitoring could be adapted to complement existing Condition-Based Monitoring (CBM) methodologies in industrial settings, improving both reliability and efficiency in predictive maintenance workflows. Future implementations of this approach could enhance early fault detection capabilities, making infrared thermography a valuable tool for real-time monitoring in high-temperature environments.

## 4. Conclusions

In this study, an image processing algorithm capable of detecting the region of interest (ROI) in the formation and growth of oxide layers in 1045 steel specimens has been implemented. All images were captured during the heating of the specimens in a controlled and standardized environment. The proposed algorithm has enabled the acquisition of specific data from the analyzed surface, demonstrating its effectiveness in accurately segmenting oxidized areas.

The acquisition of thermograms and the segmentation of oxidation areas discussed in this work are not the only possible applications of infrared thermography in this field. This process, as it can be implemented in real time, has the potential to be a valuable tool in monitoring the formation of oxide scales. This can help control the formation of oxide films at industrial levels, thereby improving quality control and efficiency in steel manufacturing processes [35].

Finally, the presented work highlights that infrared thermography, being a non-contact measurement technique, can be used to obtain information on surface reactions during the heating of steel without risk to operators. This opens new opportunities for the exploration and use of this technique in improving steel manufacturing processes in the industry. The capability to perform real-time monitoring not only increases safety but also optimizes processes, allowing for precise and timely interventions. In conclusion, this study reaffirms the significant potential of infrared thermography as an essential tool in improving steel manufacturing processes, offering substantial benefits in terms of safety, efficiency, and quality control.

## Figures and Tables

**Table 1:** Average evaluation metrics for the oxide layer segmentation algorithm.

Metric	Value
Precision	92.59%
Recall	100.00%
F1-Score	96.15%
Dice Coefficient	96.15%
IoU	92.59%
True Negative Rate	92.00%
Accuracy	96.00%

## Data Availability

The data that support the findings of this study are openly available in Zenodo at https://doi.org/10.5281/zenodo.13368190.
